# Impact of introducing endovascular treatment on acute ischemic stroke outcomes: A shift from an era of medical management to thrombectomy in Japan

**DOI:** 10.1016/j.heliyon.2020.e03945

**Published:** 2020-05-13

**Authors:** Taichiro Imahori, Junji Koyama, Kazuhiro Tanaka, Yusuke Okamura, Atsushi Arai, Hirofumi Iwahashi, Tatsuya Mori, Akiyoshi Yokote, Kazushi Matsushima, Daisaku Matsui, Makoto Kobayashi, Kohkichi Hosoda, Eiji Kohmura

**Affiliations:** a Department of Neurosurgery, Toyooka Hospital, Hyogo, Japan; b Department of Neurology, Toyooka Hospital, Hyogo, Japan; c Tajima Emergency & Critical Care Medical Center, Toyooka Hospital, Hyogo, Japan; d Department of Neurosurgery, Kobe City Nishi-Kobe Medical Center, Hyogo, Japan; e Department of Neurosurgery, Kobe University Graduate School of Medicine, Hyogo, Japan

**Keywords:** Neurology, Neurosurgery, Emergency medicine, Internal medicine, Radiology, Clinical research, Acute ischemic stroke, Large vessel occlusion, Endovascular treatment, Mechanical thrombectomy, Intravenous thrombolysis, Helicopter transport

## Abstract

**Background:**

Endovascular treatment (EVT) has increasingly become the standard treatment of acute cerebral large vessel occlusion (LVO). We evaluated the impact of introducing EVT on LVO therapy in a single center where intravenous thrombolysis (IVT) had been the only recanalization therapy.

**Materials and methods:**

Between April 2013 and March 2017, 354 consecutive patients with LVO admitted to our institution were analyzed. We compared outcomes between two chronological groups before (Pre-EVT group) and after (Post-EVT group) introducing EVT in April 2015. We assessed prognostic factors for favorable outcomes (modified Rankin scale score ≤2 at 90 days).

**Results:**

In the Pre-EVT group, all 140 patients were treated medically, including 30 patients (21%) undergoing IVT. In the Post-EVT group, 118 patients (55%) underwent EVT, and the remaining 96 patients treated medically, including six patients (3%) undergoing IVT. The proportion undergoing recanalization therapy with IVT or EVT significantly increased after introducing EVT (21% versus 58%, p < 0.001). The rate of patients achieving favorable outcomes also significantly increased (14% versus 31%, p < 0.001). In multivariate regression analysis, introducing EVT was an independent predictive factor after adjusting for age, stroke severity and extent, and time (p = 0.005). The arrival time in patients with helicopter transport was significantly shorter than that with ground ambulance for a distance of more than 10 km (p < 0.001).

**Conclusions:**

This study demonstrated that the introduction of EVT improved outcomes of acute LVO patients, increasing the opportunity to receive recanalization therapy. Further efforts to establish medical systems to provide EVT are required throughout the country.

## 1 Introduction

Acute ischemic stroke caused by large vessel occlusion (LVO) is a major public health problem and one of the leading causes of morbidity and mortality worldwide. The principal treatment approach is to recanalize the occluded vessel as soon as possible, which can improve clinical outcomes by salvaging threatened brain tissues through blood-flow restoration [1]. Intravenous thrombolysis (IVT) with tissue plasminogen activator (tPA) was the only recanalization therapy with proven efficacy in the past; however, a recent meta-analysis including five landmark randomized controlled trials (RCTs) established strong evidence for the efficacy of endovascular mechanical thrombectomy using stent retrievers over IVT [2, 3, 4, 5, 6, 7]. Since then, endovascular treatment (EVT) using stent retrievers has increasingly become the standard treatment of LVO around the world. In Japan, stent retrievers were approved for performing mechanical thrombectomy in July 2014. However, medical services for providing EVT are limited, which remains a major problem.

Our institution is a central hospital located in a rural area in Japan, covering a broad area within a radius of 70 km with the air ambulance system using helicopters. Although IVT was the only recanalization therapy in our institution in the past, we introduced the EVT approach for acute ischemic stroke therapy in April 2015. This change drastically altered the treatment measures for acute LVO in our institution at this time. Few reports have studied the changes from medical management to mechanical thrombectomy in institutions such as ours.

We evaluated the impact of introducing EVT on acute ischemic stroke therapy in a single center by comparing the outcomes of acute LVO patients before and after introducing the EVT approach. To evaluate the significance of the medical service providing EVT, we also assessed predictive factors for favorable outcomes by analyzing all patients with acute LVO.

## 2 Materials and methods

### 2.1 Patient selection

**Figure 1:**
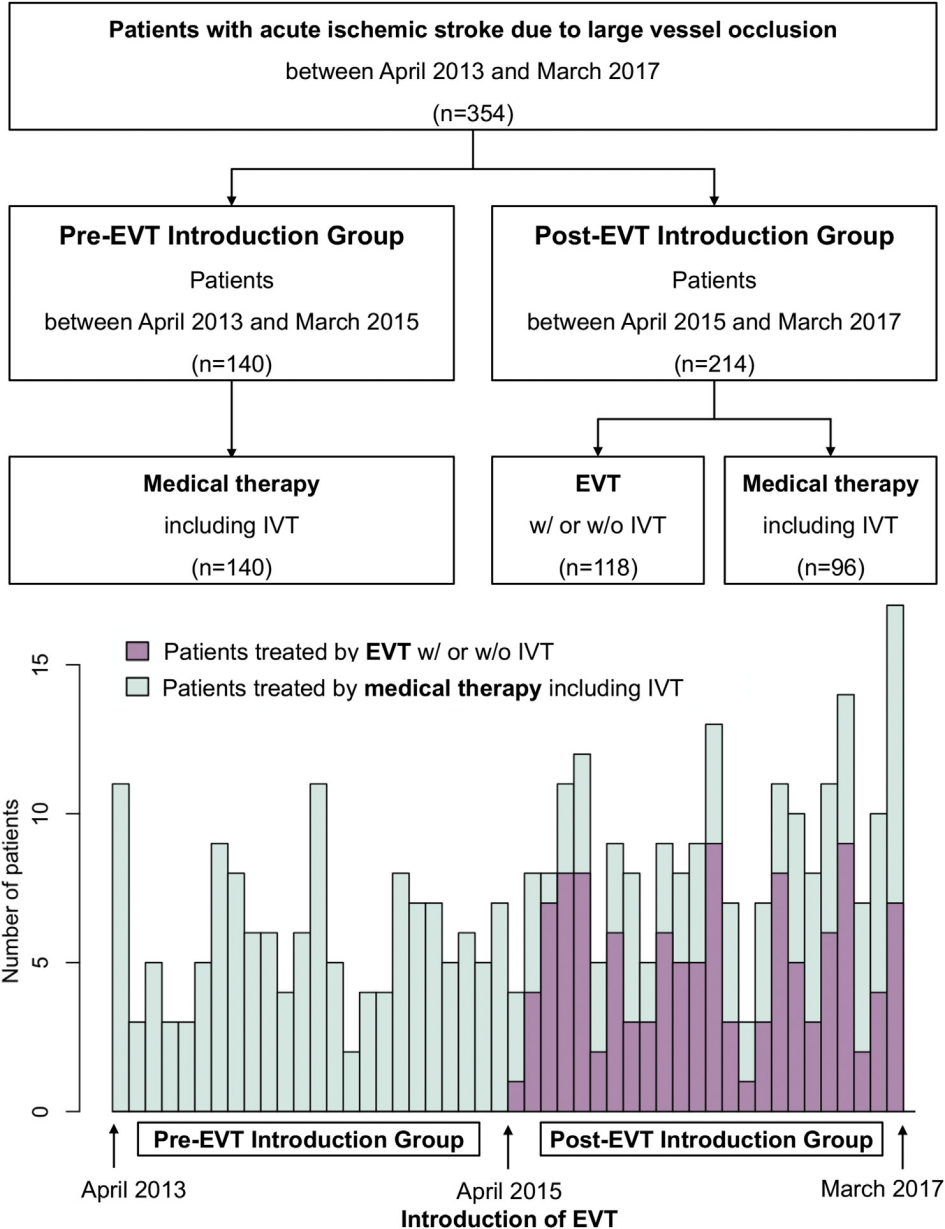
Study profile. EVT, endovascular treatment; IVT, intravenous thrombolysis; tPA, tissue plasminogen activator.

We retrospectively reviewed 354 consecutive acute ischemic stroke patients with LVO admitted to our institution in the 4 years between April 2013 and March 2017 (Figure 1). LVO was defined as acute occlusion of the vessels including the internal carotid artery (ICA), M1 and M2 portion of the middle cerebral artery (MCA), basilar artery (BA), A1 and A2 portion of the anterior cerebral artery (ACA), and P1 and P2 portion of the posterior cerebral artery (PCA), which were confirmed with computed tomography (CT) angiography or magnetic resonance angiography. We analyzed our institutional databases to examine the characteristics and treatment results. Written informed consent was obtained from a member of the patient's family before the procedure. The study design was approved by our institutional review board and was conducted in accordance with the Declaration of Helsinki.

### 2.2 Recanalization therapy

As described previously, EVT was introduced as a new recanalization therapy in April 2015 in our institution. Prior to April 2015, IVT with tPA was the only recanalization therapy; thereafter, both IVT and EVT were options for recanalization therapy. Intravenous tPA was administered within 4.5 h from stroke onset after evaluating CT or magnetic resonance images, according to the Japanese Guidelines for the Management of Stroke [8]. Our main selection criteria for patients undergoing EVT were as follows: 1) acute ischemic stroke caused by LVO as described above; 2) a score of ≥6 on the Alberta Stroke Program Early Computed Tomography Score (ASPECTS) or ASPECTS-diffusion weighted imaging; 3) certain neurological deficits as defined by a National Institutes of Health Stroke Scale (NIHSS) score ≥2; 4) <8 h from symptom onset or <24 h from the time the patient was last seen to be well in cases where the time of symptom onset was unknown. In cases treated with intravenous tPA, EVT was performed if a subsequent angiography did not show successful recanalization.

### 2.3 Endovascular procedures

Our endovascular procedures for acute LVO have been reported previously [9,10,11,12]. The Trevo stent retriever (Stryker, Kalamazoo, MI, USA) and Solitaire stent retriever (Medtronic, Minneapolis, Minnesota, USA) were used as first-line devices for endovascular mechanical thrombectomy. If a maximum of three passes of the stent retriever failed to recanalize the vessel, additional endovascular procedures, including aspiration by Penumbra catheter (Penumbra Inc., Alameda, CA, USA), percutaneous transluminal angioplasty (PTA), and intra-arterial thrombolysis using urokinase were attempted. When severe extracranial ICA stenosis was confirmed and there was difficulty with crossing the devices for mechanical thrombectomy, carotid PTA was performed before thrombectomy, and carotid artery stenting (CAS) was performed after thrombectomy if needed. Successful recanalization was defined as modified Thrombolysis in Cerebral Infarction (mTICI) score of 2b/3. Symptomatic intracranial hemorrhage was defined as subarachnoid or intracerebral hemorrhage with an increase in NIHSS score by ≥ 4 points from baseline within 24 h of the treatment.

### 2.4 Comparison between groups before and after introduction of EVT

The cohort was divided into two chronological groups: Pre-EVT introduction group (before April 2015, n = 140) and Post-EVT introduction group (after April 2015, n = 214) (Figure 1). A favorable outcome was defined as a modified Rankin scale (mRS) score ≤2 at 90 days. Baseline characteristics and treatment results for each of the two groups were compared.

### 2.5 Assessment of prognostic predictors for favorable outcomes

To evaluate the association between EVT introduction and outcomes, multivariate analyses were performed to assess prognostic predictive factors for favorable outcomes at 90 days in all patients, as described below.

### 2.6 Comparison between air ambulance and ground ambulance in group after introduction of EVT

The cohort in the Post-EVT introduction group was divided into two groups by transport method: air ambulance (helicopter transport) group (n = 80) and ground ambulance group (n = 109), with the exception of the patients who were not involved with emergency transportation system due to in-hospital stroke or other reasons. Baseline characteristics and treatment results for each of the two groups were compared, with a special focus on transport distance and arrival time from emergency call to admission.

### 2.7 Statistical analysis

Descriptive statistics are presented as the median and interquartile range (IQR). Continuous variables were compared with Welch's two-sample *t*-test and discrete variables were compared with Wilcoxon's rank-sum test. The proportions of patients with each parameter were compared using Fisher's exact test. To evaluate whether the introduction of EVT was associated with outcomes, a multivariate logistic regression analysis was conducted. The predictor variables considered were the introduction of EVT (Post-EVT introduction group) and four of the most relevant factors reported from previous studies (forced-entry method). The four selected factors as follows: age, NIHSS score on admission, ASPECTS score on admission, and time from symptom onset to admission [10, 13]. A p value < 0.05 was considered statistically significant. Statistical analysis was performed with free open-source software (R3.5.3; R Foundation for Statistical Computing; http://www.r-project.org).

## 3 Results

### 3.1 Patient characteristics

**Figure 2:**
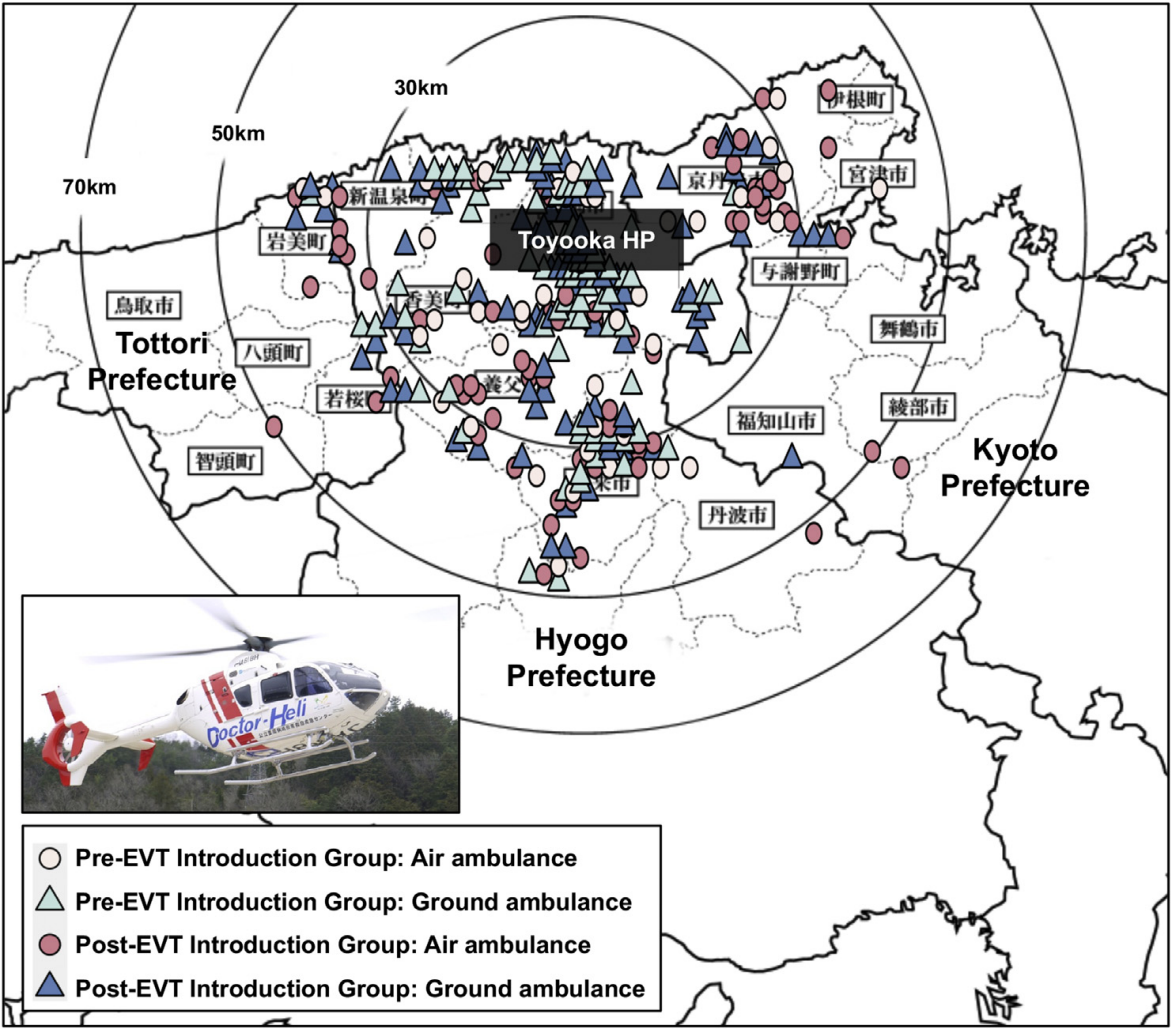
Map of areas covered by our institution. The sites where patients called for an ambulance are dotted with each symbol according to two chronological groups and two methods of transportation (air or ground ambulance). EVT, endovascular treatment.

Table 1 summarizes baseline characteristics of the 354 patients analyzed in this study, including 140 patients in the Pre-EVT introduction group and 214 patients in the Post-EVT introduction group. In the Pre-EVT introduction group, all 140 patients were treated medically. In the Post-EVT group, among 214 patients, 118 patients (55%) underwent EVT, and the remaining 96 patients (45%) were treated medically. Baseline characteristics were similar between the two groups, with the exception of transport distance. Patients in this study included many older adults with a median age of over 80 years and relatively high rate of dependency before onset in both groups. The area where patients were transported from was extended after the introduction of EVT (Figure 2). Median transport distance was 16 km (IQR = 6–24) in the Pre-EVT group and 20 km (IQR = 9–27) in the Post-EVT group (p = 0.043). Median time from emergency call to admission and from symptom onset to admission was similar in both groups, with slightly more patients transported by air ambulance in the Post-EVT introduction group (30% versus 37%, p = 0.171, respectively). There was no significant difference between the two groups in NIHSS and ASPECTS score on admission. The most common occlusion site was the MCA including M1 and M2, followed by the ICA. Other occlusion sites including the ACA, BA, and PCA were uncommon. In the Post-EVT introduction group, EVT tended to be performed in younger patients who were independent and had early admission from symptom onset, lower NIHSS score, and higher ASPECTS score.

**Table 1:** Baseline characteristics.

	Pre-EVT Introduction Group	Post-EVT Introduction Group			P Value
	Medical (n = 140)	Total (n = 214)	EVT (n = 118)	Medical (n = 96)	
Age (years)	83 (74–87)	83 (74–89)	80 (72–85)	86 (80–91)	0.352
Male	69 (49%)	102 (48%)	66 (56%)	36 (38%)	0.828
mRS ≤2 before stroke onset	102 (73%)	164 (77%)	105 (89%)	59 (61%)	0.452
Transport distance (km)	16 (6–24)	20 (9–27)	20 (10–27)	20 (9–27)	0.043∗
Air ambulance (helicopter transport)	42 (30%)	80 (37%)	47 (40%)	33 (34%)	0.171
Time from emergency call to admission (min)	43 (31–57)	42 (32–60)	41 (32–61)	43 (34–59)	0.165
Time from symptom onset to admission (min)	203 (77–591)	170 (83–479)	125 (77–330)	228 (95–626)	0.201
NIHSS score on admission	19 (13–27)	19 (9–25)	15 (8–23)	21 (9–27)	0.067
ASPECTS score on admission	7 (5–9)	8 (5–9)	8 (7–10)	5 (2–8)	0.432

Occlusion site					

Internal carotid artery	34 (24%)	66 (31%)	32 (27%)	34 (35%)	0.187
M1 segment middle cerebral artery	57 (41%)	78 (36%)	51 (43%)	27 (28%)	0.435
M2 segment middle cerebral artery	27 (19%)	45 (21%)	23 (19%)	22 (23%)	0.787
A1 or A2 segment anterior cerebral artery	4 (3%)	3 (1.4%)	1 (1%)	2 (2%)	0.441
Basilar artery	12 (9%)	11 (5%)	5 (4%)	6 (6%)	0.270
P1 or P2 segment posterior cerebral artery	6 (4%)	11 (5%)	6 (5%)	5 (5%)	0.803

Data are presented as median (interquartile range) or number (%).

EVT, endovascular treatment; mRS, modified Rankin scale; NIHSS, National Institutes of Health Stroke Scale; ASPECTS, Alberta Stroke Program Early Computed Tomography Score.

∗ Statistically significant.

### 3.2 Endovascular treatment

The results of endovascular treatment are summarized in Table 2. The most common procedure was mechanical thrombectomy using stent retrievers, which was performed in 111 patients (94%). Other endovascular procedures including mechanical thrombectomy using aspiration catheter, intracranial PTA, intra-arterial thrombolysis, and CAS were also performed with mechanical thrombectomy using stent retrievers in some cases. Finally, successful recanalization (mTICI 2b/3) was achieved in 103 patients (87%), with median time from admission to groin puncture of 91 min and time from puncture to recanalization of 36 min.

**Table 2:** Results of endovascular treatment.

	Post-EVT Introduction Group
	EVT (n = 118)
Endovascular procedure	

Mechanical thrombectomy using stent retriever	111 (94%)
Mechanical thrombectomy using aspiration catheter	14 (12%)
Intracranial PTA	11 (9%)
Intra-arterial thrombolysis	17 (14%)
CAS	7 (6%)
Successful recanalization (mTICI 2b/3)	103 (87%)
Time from admission to groin puncture (min)	91 (82–109)
Time from puncture to recanalization (min)	36 (26–46)
Symptomatic intracranial hemorrhage	7 (6%)

Data are presented as number (%).

EVT, endovascular treatment; PTA, percutaneous transluminal angioplasty; CAS, carotid artery stenting; mTICI, modified Thrombolysis in Cerebral Infarction.

### 3.3 Overall treatment results and outcomes

**Figure 3:**
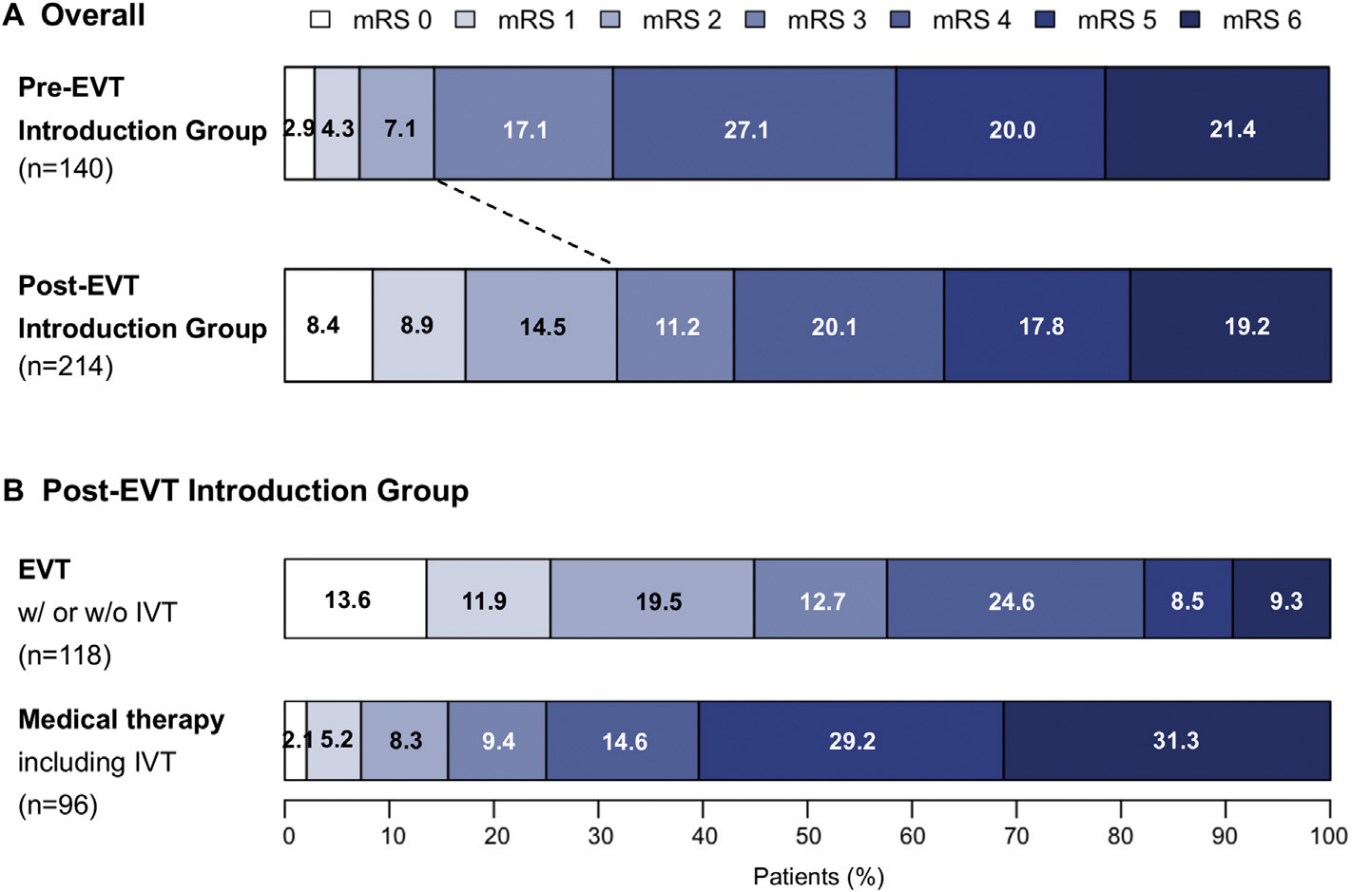
Scores on the modified Rankin Scale at 90 days. (A) Overall. (B) Post-EVT introduction group. EVT, endovascular treatment IVT; intravenous thrombolysis; tPA, tissue plasminogen activator; mRS, modified Rankin scale.

Treatment results and outcomes are summarized in Table 3. In the Pre-EVT group, IVT with tPA was performed in 30 patients (21%). In the Post-EVT group, IVT and EVT were performed in 29 (14%) and 118 patients (55%), respectively, resulting in 124 patients (58%) who received recanalization therapy with IVT, EVT, or both. Accordingly, the proportion of patients receiving recanalization therapy was significantly increased after the introduction of EVT (21% versus 58%, p < 0.001, respectively). Significantly more patients with favorable outcomes (mRS ≤2) at 90 days in the era after the introduction of EVT (14% versus 31%, p < 0.001, respectively) (Figure 3) were observed.

**Table 3:** Treatment results and outcomes.

	Pre-EVT Introduction Group	Post-EVT Introduction Group			P Value
	Medical (n = 140)	Total (n = 214)	EVT (n = 118)	Medical (n = 96)	
Revascularization therapy with IVT or EVT (IVT alone + EVT)	30 (21%)	124 (58%)	118 (100%)	6 (6%)	<0.001∗
IVT (intravenous tPA)	30 (21%)	29 (14%)	23 (19%)	6 (6%)	0.059
IVT alone	30 (21%)	6 (3%)	-	6 (6%)	-
Both IVT and EVT	-	23 (11%)	23 (19%)	-	-
EVT	-	118 (55%)	118 (100%)	-	-
Favorable outcome (mRS ≤2 at 90 days)	20 (14%)	67 (31%)	52 (44%)	15 (16%)	<0.001∗
Death (mRS 6 at 90 days)	30 (21%)	41 (19%)	11 (9%)	30 (31%)	0.684

Data are presented as number (%).

EVT, endovascular treatment; IVT, intravenous thrombolysis; tPA, tissue plasminogen activator; mRS, modified Rankin scale.

∗ Statistically significant.

### 3.4 Prognostic predictive factors

Table 4 shows the results of multivariate analyses of prognostic factors predicting outcomes at 90 days in all patients with LVO. Multivariate regression analysis revealed that all five factors were independently associated with outcomes. The introduction of EVT (Post-EVT introduction group) was the most influential predictive factor for favorable outcomes, with an odds ratio of 2.95 (95% confidence interval, 1.41–6.46; p = 0.005).

**Table 4:** Prognostic predictive factors for outcome.

	Univariate			Multivariate	
	Favorable outcome (mRS ≤2) (n = 87)	Unfavorable outcome (mRS ≥3) (n = 267)	P Value	Odds ratio (95% CI)	P Value
Introduction of EVT (Post-EVT introduction group)	67 (77%)	147 (55%)	<0.001∗	2.95 (1.41–6.46)	0.005∗
Age (years)	75 (71–84)	84 (77–89)	<0.001∗	0.98 (0.83–0.96)	0.003∗
NIHSS score on admission	8 (4–15)	21 (14–27)	<0.001∗	0.89 (0.84–0.93)	<0.001∗
ASPECTS score on admission	9 (8–10)	7 (4–8)	<0.001∗	1.41 (1.16–1.75)	0.001∗
Time from symptom onset to admission (min)	114 (69–282)	204 (83–626)	<0.001∗	0.90 (0.83–0.96)	0.005∗

Data are presented as median (interquartile range) or number (%).

mRS, modified Rankin scale; CI, confidence interval; EVT, endovascular treatment; NIHSS, National Institutes of Health Stroke Scale; ASPECTS, Alberta Stroke Program Early Computed Tomography Score.

The odds of age, NIHSS score on admission, and ASPECTS score on admission are presented as odds per one increase. The odds of time from stroke onset to admission are presented as odds per 60-min increase.

∗ Statistically significant.

### 3.5 Comparison between air ambulance and ground ambulance in group after introduction of EVT

**Figure 4:**
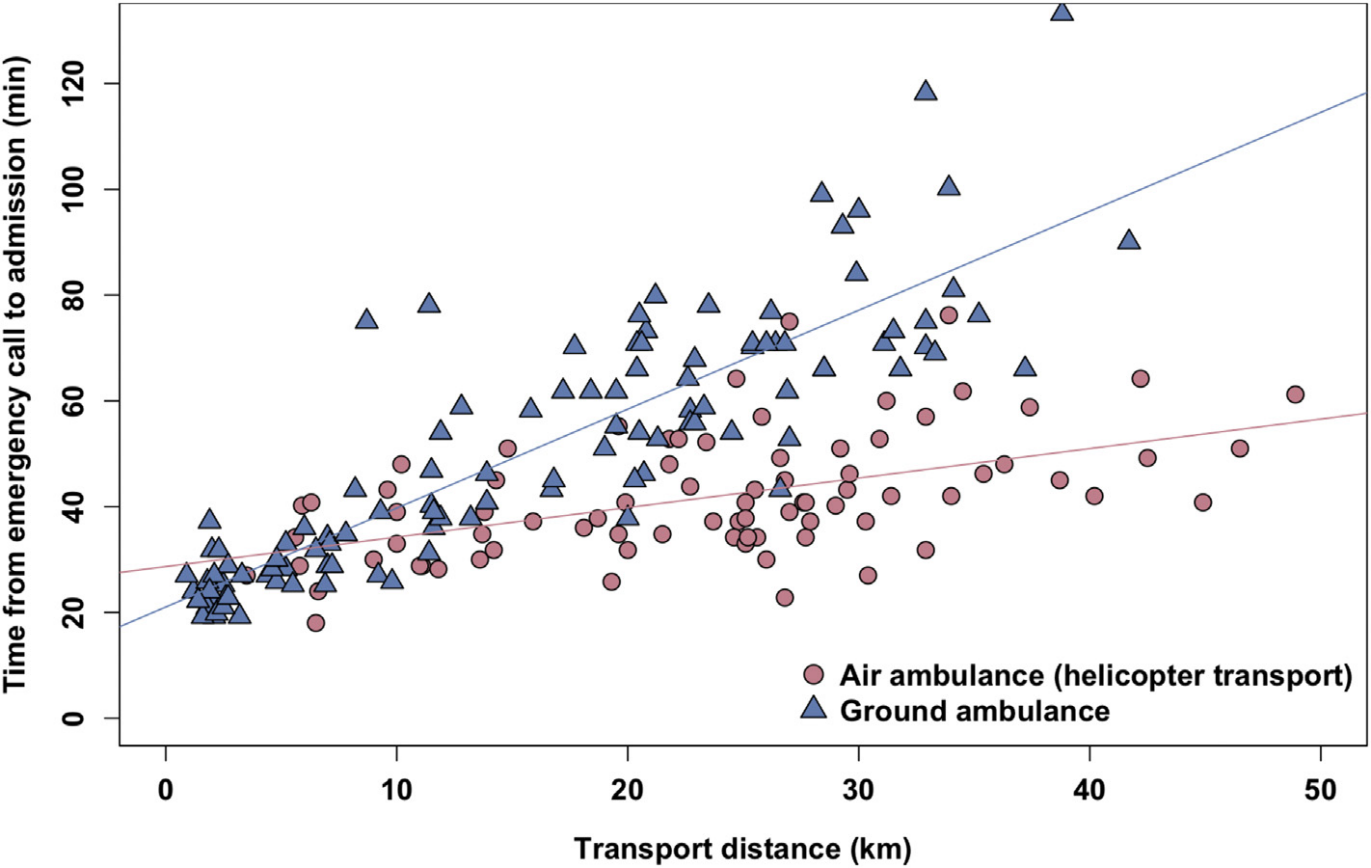
Time from emergency call to admission and transport distance according to method of transportation.

**Figure 5:**
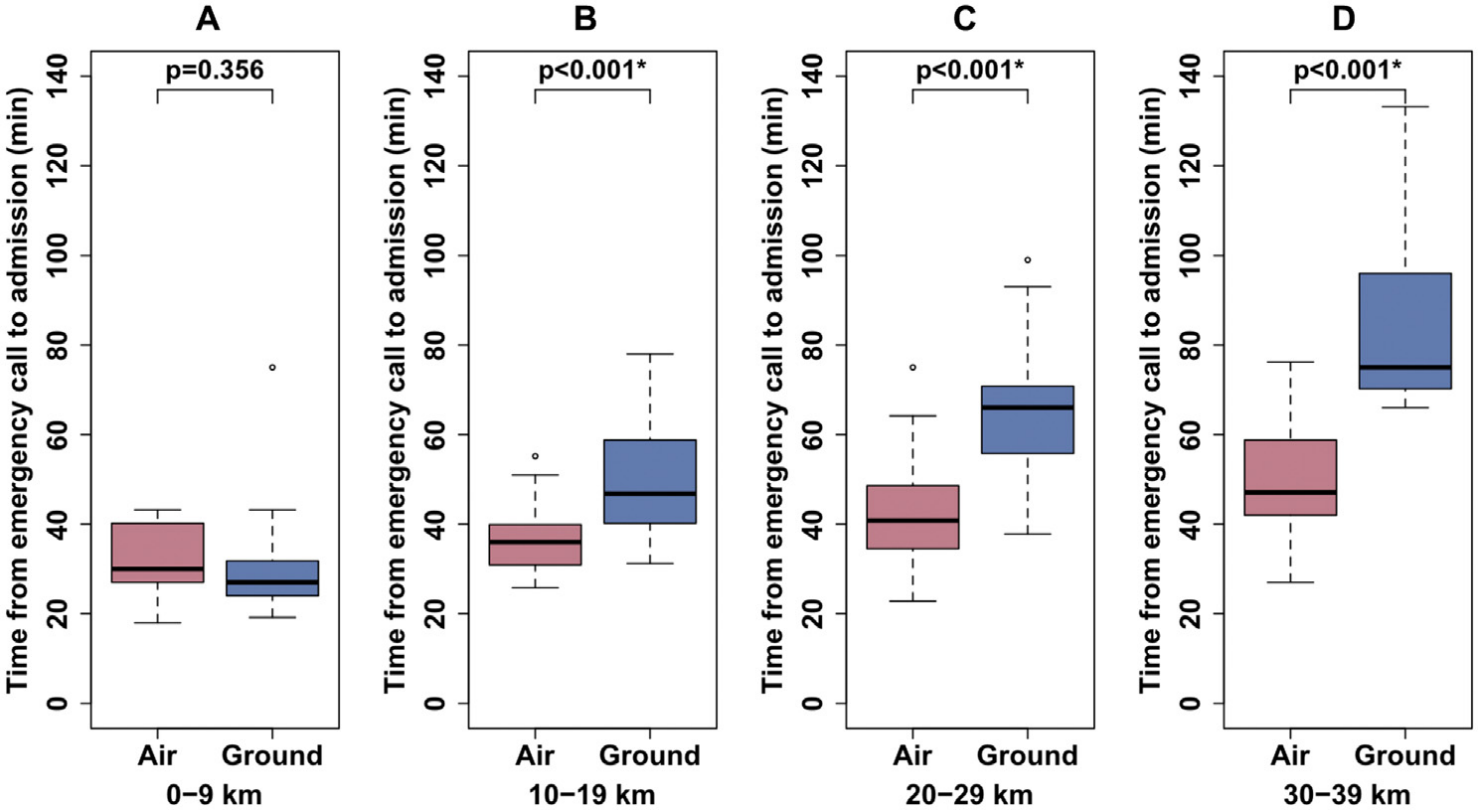
Time from emergency call to admission according to method of transportation and transport distance. (A) Transport distance: 0–9 km. (B) Transport distance: 10–19 km. (C) Transport distance: 20–29 km. (D) Transport distance: 30–39 km. Air, air ambulance (helicopter transport); Ground, ground ambulance. ∗Statistically significant.

Table 5 summarizes baseline characteristics and treatment results of the 80 patients in the air ambulance (helicopter transport) group and 109 patients in the ground ambulance group. Baseline characteristics were similar between the two groups, with the exception of transport distance and time from emergency call to admission. Median transport distance in the air ambulance group was significantly longer than that in the ground ambulance group (25 km versus 17 km, p < 0.001, respectively). However, median time from emergency call to admission in the air ambulance group was significantly shorter than that in the ground ambulance group (41min versus 47min, p < 0.001, respectively), and treatment results were similar between the two groups. Figures 4 and 5 shows scatter plots and boxplots of time from emergency call to admission according to method of transportation and transport distance. For a distance of more than 10 km, median time from emergency call to admission in the air ambulance group was significantly shorter than that in the ground ambulance group (p < 0.001).

**Table 5:** Comparison between air ambulance and ground ambulance in group after introduction of EVT.

	Air ambulance (helicopter transport) (n = 80)	Ground ambulance (n = 109)	P Value
Age (years)	83 (76–89)	83 (75–89)	0.568
NIHSS score on admission	20 (11–25)	17 (9–26)	0.787
ASPECTS score on admission	8 (4–10)	7 (6–9)	0.899
Transport distance (km)	25 (15–30)	17 (6–25)	<0.001∗
Time from onset to emergency call (min)	95 (19–454)	105 (26–342)	0.656
Time from emergency call to admission (min)	41 (34–48)	47 (29–70)	<0.001∗
Transport distance: 0–9 km	30 (27–40)	27 (24–32)	0.356
Transport distance: 10–19 km	36 (31–40)	47 (40–59)	<0.001∗
Transport distance: 20–29 km	41 (35–48)	66 (56–71)	<0.001∗
Transport distance: 30–39 km	47 (42–58)	75 (70–96)	<0.001∗
Time from onset to admission (min)	134 (66–491)	175 (89–396)	0.781
Revascularization therapy with IVT or EVT	50 (63%)	60 (55%)	0.371
IVT (intravenous tPA)	14 (18%)	14 (13%)	0.411
EVT	47 (57%)	57 (52%)	0.460
Favorable outcome (mRS ≤2 at 90 days)	24 (30%)	34 (31%)	0.875
Death (mRS 6 at 90 days)	13 (16%)	23 (21%)	0.457

Data are presented as median (interquartile range) or number (%).

EVT, endovascular treatment; mRS, modified Rankin scale.

NIHSS, National Institutes of Health Stroke Scale; ASPECTS, Alberta Stroke Program Early Computed Tomography Score; IVT, intravenous thrombolysis; tPA, tissue plasminogen activator; mRS, modified Rankin scale.

∗ Statistically significant.

## 4 Discussion

This study demonstrated that the introduction of EVT significantly improved outcomes of acute ischemic stroke patients with LVO, substantially increasing the opportunity to receive recanalization therapy. After introducing EVT, approximately 55% of patients in this study underwent EVT. This study also revealed that the introduction of EVT was a significant predictive factor for favorable outcomes in patients with acute LVO. The drastic shift from an era of medical management to thrombectomy demonstrated the overall effect of EVT on acute ischemic therapy in a real-world setting. Our study also showed that helicopter emergency transportation system could achieve prompt arrival in our broad rural area.

This study demonstrated that the introduction of EVT significantly improved overall outcomes of acute ischemic stroke patients with LVO, including patients treated without EVT. Globally, EVT has become the standard treatment for LVO with proven efficacy since the advent of mechanical thrombectomy using stent retrievers. A meta-analysis of five recent RCTs, the Highly Effective Reperfusion evaluated in Multiple Endovascular Stroke Trials (HERMES) study, has shown a strong benefit of EVT over standard medical treatment with nearly twofold improvement of outcomes [2]. However, few reports exist on the overall effect of introducing the EVT approach on acute ischemic therapy in real-world settings. By comparing the outcomes of acute LVO patients before and after introducing the EVT approach, this study demonstrated that the introduction of EVT was highly beneficial, resulting in approximately twofold improvement of favorable outcomes, reflecting the change from an era of medical management to mechanical thrombectomy (14% of mRS ≤2 at 90 days in the Pre-EVT group versus 31% in the Post-EVT group, p < 0.001).

The main factor altered before and after introducing the EVT approach in this study was the increased proportion of patients receiving recanalization therapy. Before introducing EVT, IVT was previously the only recanalization therapy and was performed in only 21% of patients with acute LVO. After introducing EVT, EVT was performed with or without IVT in 55% of patients, and IVT alone was performed in only 3% of patients. The increased opportunity to receive recanalization therapy by introducing EVT was primarily due to the therapeutic time limit for EVT. According to a previous study for geographic disparities in tPA use for acute ischemic stroke, a rural-urban disparity was evident, with fourfold higher rates in urban hospitals than in rural hospitals [14]. In broad rural areas such as those covered by our institution, the time from onset to hospital arrival tends to be insufficient for the time limit of undergoing IVT. However, the longer therapeutic time limit of EVT could increase the opportunity to receive recanalization therapy, as indicated in this study.

This study also revealed that the introduction of EVT was a significant predictive factor for favorable outcomes in patients with acute LVO. Age, initial stroke severity represented by baseline NIHSS and ASPECTS, and time from stroke onset are well-known predictive factors for outcomes in patients with acute LVO [10, 13]. In this study, multivariate logistic regression using these four factors and the introduction of EVT (Post-EVT introduction group) revealed that all five factors were significant independent predictive factors for outcomes. Moreover, the introduction of EVT was the most influential factor for favorable outcomes, with an odds ratio of 2.95 (95% confidence interval, 1.41–6.46; p = 0.005). Collectively, these findings emphasize the overall beneficial effects of EVT on acute ischemic therapy in a real-world setting.

Mechanical thrombectomy using stent retrievers was performed as the main endovascular procedure in most cases in this study. Final successful recanalization (mTICI 2b/3) was achieved in 87% of cases, with a favorable outcome rate of 44%. The results of EVT in this study were almost comparable to those of previous studies including RCTs and in real-word settings, although the portion of the patients with M2 occlusion was somewhat high in our study [3, 4, 5, 6, 7, 15, 16]. The patients who underwent EVT in this study tended to be younger and independent, with early admission from symptom onset, lower NIHSS score, and higher ASPECTS score compared to those who did not undergo EVT. Recent RCTs have indicated the efficacy of the expanded therapeutic time limit for EVT [17, 18]. Further indications for EVT for patients such as those with low initial ASPECTS score and distal occlusions have also been reported [19]. Increasing use of EVT may occur for acute ischemic stroke therapy in the future.

Despite the clinical benefits of EVT shown to date, a major remaining issue is that the medical service for providing EVT is limited, especially in rural areas. Our institution is a central hospital located in a rural area in Japan, covering a broad area including mountainous regions. In this study, the median transport distance was significantly increased after introducing EVT, with an approximately 1.5-fold increase in the total patients admitted to our institute and an increased use of the helicopter transport. In addition, our study showed that air ambulance using helicopter could provide prompt transportation in our broad rural area. For a distance of more than 10 km, the arrival time in patients with helicopter transport was significantly shorter than that with ground ambulance for a distance of more than 10 km (p < 0.001). Previous studies have shown that helicopter transport may contribute to shorter hospital arrival time and higher thrombolysis rates in acute ischemic stroke patients [20, 21]. Helicopter transport would also contribute to the use of EVT for acute ischemic patients in rural areas. The results of this study indicate a trend towards the centralization of stroke patients to an endovascularly treatable facility utilizing emergency system including helicopter transport. A major contributor to this centralization of stroke patients may be our continuous campaigns for enlightening the community residents and ambulance crews on stroke awareness and knowledge of the importance of the early treatment. These movements for reinforcement of regional medical service should be important in the future, especially in rural areas. Further efforts to establish medical systems to provide EVT are needed throughout the country.

Limitations of this study include the small sample size, which had low power for detecting significant differences, and the retrospective nature of the analysis, which lacked blinding between groups. Nevertheless, the latter is both a weakness and a strength because our analysis of patients before and after the introduction of EVT highlights the benefits of introducing the EVT approach on acute ischemic therapy in a real-world setting.

## 5 Conclusions

This study demonstrated that the introduction of EVT significantly improved outcomes of acute ischemic stroke patients with LVO, substantially increasing the opportunity to receive recanalization therapy. The findings from this drastic shift from an era of medical management to that of thrombectomy highlight the overall effect of EVT on acute ischemic therapy in a real-world setting. Our study also showed that air ambulance system using helicopter could provide prompt transportation, which may make tremendous contributions to acute ischemic stroke therapy, especially in broad rural areas. Further efforts to establish medical systems for EVT provision are needed throughout the country.

## Declarations

### Author contribution statement

T. Imahori: Conceived and designed the experiments; Performed the experiments; Analyzed and interpreted the data; Contributed reagents, materials, analysis tools or data; Wrote the paper.

J. Koyama, K. Tanaka, Y. Okamura, A. Arai, H. Iwahashi, T. Mori, A. Yokote, K. Matsushima, D. Matsui and M. Kobayashi: Performed the experiments.

K. Hosoda and E. Kohmura: Analyzed and interpreted the data.

### Funding statement

A part of this research was supported by Grants-in-Aid for Scientific Research JSPS KAKENHI (Grant Number JP20K17968) and Medical Research Fund of Hyogo Medical Association (Grant Number MRF-H-04-17).

### Competing interest statement

The authors declare no conflict of interest.

### Additional information

No additional information is available for this paper.
